# Nonalcoholic Fatty Liver Disease and Insulin Resistance: New Insights and Potential New Treatments

**DOI:** 10.3390/nu9040387

**Published:** 2017-04-14

**Authors:** Hironori Kitade, Guanliang Chen, Yinhua Ni, Tsuguhito Ota

**Affiliations:** Department of Cell Metabolism and Nutrition, Brain/Liver Interface Medicine Research Center, Kanazawa University, Kanazawa, Ishikawa 920-8640, Japan; hiro.kitacchi@gmail.com (H.K.); guanliangc@gmail.com (G.C.); shali0145@gmail.com (Y.N.)

**Keywords:** NAFLD/NASH, macrophage/Kupffer cells, chemokine, insulin resistance, inflammation, fibrosis, antioxidants, astaxanthin, β-cryptoxanthin

## Abstract

Nonalcoholic fatty liver disease (NAFLD) is one of the most common chronic liver disorders worldwide. It is associated with clinical states such as obesity, insulin resistance, and type 2 diabetes, and covers a wide range of liver changes, ranging from simple steatosis to non-alcoholic steatohepatitis (NASH), liver cirrhosis, and hepatocellular carcinoma. Metabolic disorders, such as lipid accumulation, insulin resistance, and inflammation, have been implicated in the pathogenesis of NAFLD, but the underlying mechanisms, including those that drive disease progression, are not fully understood. Both innate and recruited immune cells mediate the development of insulin resistance and NASH. Therefore, modifying the polarization of resident and recruited macrophage/Kupffer cells is expected to lead to new therapeutic strategies in NAFLD. Oxidative stress is also pivotal for the progression of NASH, which has generated interest in carotenoids as potent micronutrient antioxidants in the treatment of NAFLD. In addition to their antioxidative function, carotenoids regulate macrophage/Kupffer cell polarization and thereby prevent NASH progression. In this review, we summarize the molecular mechanisms involved in the pathogenesis of NAFLD, including macrophage/Kupffer cell polarization, and disturbed hepatic function in NAFLD. We also discuss dietary antioxidants, such as β-cryptoxanthin and astaxanthin, that may be effective in the prevention or treatment of NAFLD.

## 1. Introduction

**Figure 1:**
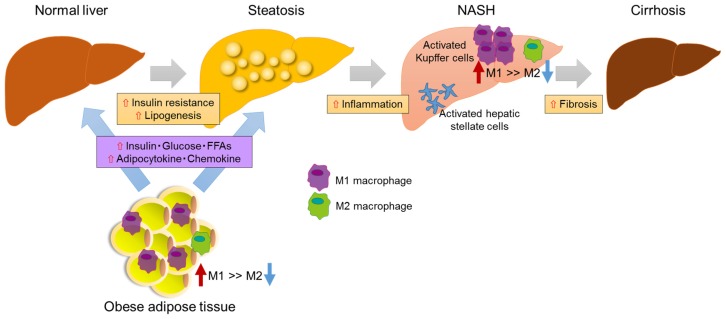
Hypothesis explaining the progression of NAFLD/NASH. Overnutrition or inactivity leads to adipocyte hypertrophy and dysfunction, which are linked to chronic inflammation and insulin resistance through the recruitment and activation of immune cells such as macrophages and T-cells. Excess fat intake and obesity lead to hyperglycemia, hyperlipidemia, and the oversecretion of adipocytokines and the chemokines tumor necrosis factor (TNF)-α, interleukin (IL)-1β, and monocyte chemoattractant protein (MCP)-1/C-C chemokine ligand 2 (CCL2). These factors further contribute to the development of systemic insulin resistance and hepatic steatosis. The latter causes hepatic inflammation and induces NASH and even cirrhosis. Hepatic inflammation involves the recruitment of macrophages/Kupffer cells and an M1-dominant phenotypic shift in macrophages in the liver, activating hepatic stellate cells and finally leading to liver fibrosis.

Nonalcoholic fatty liver disease (NAFLD) is one of the most important chronic liver disorders worldwide [1]. It covers a wide spectrum of hepatic damage in which steatosis with inflammation progresses to non-alcoholic steatohepatitis (NASH), fibrosis, cirrhosis, and, ultimately, hepatocellular carcinoma [2,3,4,5]. NAFLD is considered to be the hepatic component of metabolic syndrome as its features are similar to those of metabolic disorders such as obesity, inflammation, insulin resistance, and type 2 diabetes [6,7,8]. Thus, it is important to treat NAFLD as well as its associated metabolic diseases [9,10,11]. However, the mechanisms underlying the pathogenesis and progression of NAFLD are still incompletely understood. The two-hit-hypothesis has been proposed to explain the pathogenesis of NASH [12]. The first hit is insulin resistance and excessive fatty acids in the circulation, which lead to simple hepatic steatosis (Figure 1). We previously showed that insulin resistance promoted the progression from simple fatty liver to NASH [13]. The second hit involves oxidative stress, lipid peroxidation, and mitochondrial dysfunction. With the identification of more advanced mechanisms, NASH was shown to develop through a multifactorial process that includes insulin resistance, oxidative stress, genetic determinants, nutrition and lifestyle, endoplasmic reticulum stress, inflammation, and changes in the intestinal microbiota [14].

Insulin resistance is pivotal for the progression of NAFLD [6]. It has been shown that NAFLD is closely associated with insulin resistance, as 70%–80% of obese and diabetic patients have NAFLD [8,15]. Immune cells, macrophages/Kupffer cells, natural killer cells, and T-cells contribute to the progression of NASH and their potential therapeutic targets. In particular, hepatic macrophages, which include both resident Kupffer cells and recruited bone marrow-derived macrophages, are the major immune cells that secrete inflammatory mediators, such as tumor necrosis factor (TNF)-α and interleukin (IL)-1β, leading to systemic insulin resistance and NASH [16]. Macrophages can be classified as M1, or “classically activated” pro-inflammatory macrophages, and M2, or “alternatively activated” non-inflammatory macrophages [17,18,19]. Alternative M2 macrophages sustain insulin sensitivity via the secretion of anti-inflammatory cytokines such as IL-4 and IL-13, while classical M1 macrophages secrete pro-inflammatory cytokines such as TNF-α, IL-6, and IL-1β, which, in turn, leads to insulin resistance and NASH [18,19]. Thus, the dysregulation and polarization of M1 and M2 macrophages are closely related to multiple metabolic disorders, among them, obesity, insulin resistance, and NAFLD. Previously, we found that excessive hepatic lipid accumulation promoted the activation of macrophages/Kupffer cells to exacerbate insulin resistance, as well as hepatic inflammation and fibrogenesis [20].

Although oxidative stress is also closely associated with the progression of NASH, there is no established standard therapy for this disease [21,22,23]. Metformin, thiazolidinedione, and liraglutide are among the therapeutic agents currently used for the treatment of NASH [24,25,26]. However, in the TONIC trial, neither metformin nor vitamin E improved the liver histology in NASH patients; there was also no sustained reduction in alanine aminotransferase (ALT) levels in children and adolescents with NAFLD. Although the NASH resolution was greater in vitamin-E-treated patients, fibrosis was not improved [25]. In the PIVENS trial, pioglitazone and vitamin E improved hepatic steatosis and lobular inflammation in NASH, but not the fibrosis scores [24]. In the LEAN trial, liraglutide led to the histological resolution of NASH [26]. Thus, the need for effective pharmacological therapies remains, although bariatric surgery is curative for NAFLD/NASH [27]. In this review, we discuss the association between hepatic macrophages/Kupffer cells and NASH pathogenesis. We also examine several potentially effective micronutrient antioxidants that hold promise in NAFLD prevention and therapy.

## 2. Pathogenesis of NAFLD

### 2.1. Insulin Resistance and NAFLD

Obesity leads to NAFLD development through liver dysfunction caused by hepatic steatosis. In obese patients with concomitant type 2 diabetes and NAFLD, hyperinsulinemia and dyslipidemia are more severe than in patients without NAFLD [28]. Excess fatty acids, the production of which is induced by lipogenesis and fatty acid synthesis, as well as oxidated fatty acids, circulate in peripheral tissues, including liver and adipose tissue, where they accumulate, resulting in insulin resistance [29]. Adipose tissue is a mediator of systemic lipid storage, as well as an endocrine organ that secretes hormones and the group of cytokines known as adipokines, such as adiponectin and leptin [30]. Adiponectin is a specific secretory adipokine that regulates fatty acid oxidation and inhibits lipid accumulation, both in adipose tissue and in the liver [31]. It also maintains whole-body glucose homeostasis, including hepatic insulin sensitivity [32]. Recent studies have shown that serum adiponectin levels are lower in patients with than without NAFLD [33]. Hypoadiponectinemia in the development of NAFLD or type 2 diabetes impairs fatty acid metabolism and promotes a chronic inflammatory state in the liver [34,35]. Thus, the maintenance of adiponectin levels may prevent patients with NAFLD from developing inflammation and fibrosis.

### 2.2. Macrophages/Kupffer Cells

Overnutrition or insufficient exercise leads to adipose expansion, with the hypertrophic adipocytes secreting TNF-α, IL-1β, and IL-6. These pro-inflammatory cytokines down-regulate hepatic insulin sensitivity via the activation of pro-inflammatory signaling and the inhibition of insulin receptor signaling. The result is the development of liver steatosis and fibrosis [36]. In the liver, resident macrophages/Kupffer cells are central players in the development of NASH, by recruiting inflammatory immune cells and secreting pro-inflammatory cytokines [37]. These cells localize within liver sinusoids, accounting for ~10% of the total number of liver cells [38]. The macrophage markers F4/80, CD11b, and CD68 are expressed on Kupffer cells. Those in the liver are F4/80^+^CD68^+^ phagocytic macrophages that produce reactive oxygen species. Cytokine-producing bone-marrow-derived macrophages express CD11b [39]. In contrast to CD11b^+^ cells, CD68^+^ cells preferentially adhere to liver sinusoidal endothelial cells or hepatocytes [40,41].

In response to various signals, macrophages may undergo classical M1 activation, in which they are stimulated by toll-like receptor ligands and interferon-γ, or alternative M2 activation, in which they are stimulated by IL-4/IL-13 [42,43]. Inflammation in the liver is regulated by the balance of pro-inflammatory M1 Kupffer cells and anti-inflammatory M2 Kupffer cells [37]. Thus, the exacerbated release of M1 Kupffer-cell-derived mediators contributes to the pathogenesis of liver steatosis, the recruitment of inflammatory immune cells, and the activation of fibrogenesis [37,44]. Inflammatory cytokines, which in addition to TNF-α include chemokines such as monocyte chemoattractant protein (MCP)-1/C-C chemokine ligand 2 (CCL2) and RANTES/CCL5, are produced by M1 Kupffer cells and increase hepatic lipid accumulation, which results in the discordant regulation of lipid metabolism and homeostasis [45]. In contrast, the alternative activation of M2 Kupffer cells is a critical pathway for the resolution of inflammatory responses in NAFLD. M2 Kupffer cells promote the caspase-3-dependent apoptosis of classically activated M1 Kupffer cells and thus provide a protective mechanism against NAFLD [44]. Because the M1/M2 ratio is increased during NAFLD progression, the polarization of cells into M2 Kupffer cells might be an important mechanism protecting against fatty liver disease.

### 2.3. Role of Chemokine in NAFLD

Chemokines are a family of cytokines that activate leukocyte chemotaxis and play important roles in the progression of systemic inflammation [46,47]. By recruiting immune cells to adipose tissue, liver, and skeletal muscle, chemokines lead to acute inflammation and the development of insulin resistance, as well as fatty liver disease [48,49,50]. MCP-1, also known as CCL2, is up-regulated in obese adipose tissue, secondary to macrophage infiltration [51,52]. By binding to the CCR2 receptor, MCP-1 causes the infiltration of bone marrow-derived macrophages into obese adipose tissue or liver. It is also involved in the development of hepatic steatosis and insulin resistance [50,51,52]. Indeed, the specific overexpression of MCP-1 in the adipose tissue of mice leads to their development of insulin resistance, inflammation, and hepatic steatosis [53]. Conversely, mice with a genetic deletion of CCR2 have improved insulin sensitivity and inflammation, without a decrease in body weight, in agreement with the findings obtained with pharmacological antagonists of CCR2 [51,54]. Therefore, by recruiting macrophages, MCP-1-CCR2 signaling plays a central role in the development of inflammation and insulin resistance.

**Figure 2:**
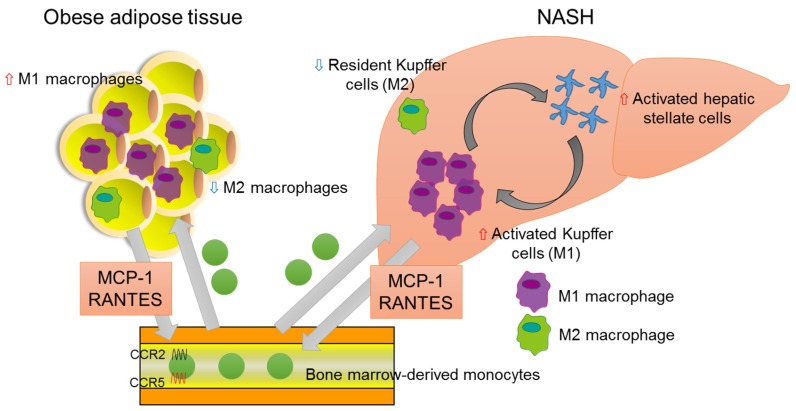
Association of chemokines and NASH. In adipose tissue of the obese, bone-marrow-derived monocytes are recruited from the bloodstream, predominantly via MCP-1-CCR2 signaling. The RANTES-CCR5 pathway also plays an important role in monocyte recruitment in adipose tissue. Infiltrated macrophages in obese adipose tissue undergo a phenotypic switch from alternative M2 macrophages to classical M1 macrophages. The latter secrete pro-inflammatory cytokines, which result in insulin resistance, adipokine dysfunction, and excess lipid accumulation in the liver. In the fatty liver, the recruitment and activation of immune cells, including Kupffer cells, contribute to hepatic inflammation, which is involved in hepatic stellate cell activation.

However, there are also studies reporting conflicting results, showing that MCP-1-deficient mice do not exhibit reduced macrophage infiltration or improved insulin sensitivity, which suggests that MCP-1-CCR2 signaling is not critical for obesity-induced macrophage recruitment or systemic insulin resistance [55,56]. Instead, other chemokines involved in obesity may contribute to macrophage recruitment and insulin resistance. In previous work, we demonstrated that CCR5-deficient mice are protected from insulin resistance and hepatic fatty acid infiltration, through the regulation of macrophage recruitment and the response of M1/M2 macrophage polarization to inflammation (Figure 2) [57].

The hepatic infiltration of macrophages/Kupffer cells is primarily promoted by MCP-1, as these cells express CCR2 [58]. MCP-1 expression in hepatocytes is increased in animals fed a high-fat diet and leads to the hepatic recruitment of CCR2^+^ myeloid cells that promote hepatic steatosis [50]. The MCP-1-CCR2 pathway is also up-regulated in the livers of animals with NASH and is thus critical to the development of hepatic steatosis and fibrosis by promoting the migration of hepatic stellate cells [59,60]. In fact, serum and liver MCP-1 levels are increased in NASH patients [58], whereas in animal NASH models, the genetic deletion of MCP-1 and CCR2 or the inactivation of CCR2 reduces macrophage infiltration, attenuates obesity, and improves both insulin resistance and hepatic steatosis [51,52,59]. Thus, collectively, MCP-1-CCR2 signaling is central to the progression of hepatic steatosis to NASH.

RANTES/CCL5 binds to CCR1, CCR3, and CCR5 to promote the migration of T-cells, monocytes, neutrophils, and dendritic cells. Both CCR5 and its ligands are overexpressed in a dietary model of hepatic steatosis [57,61]. In NAFLD and NASH, hepatocytes are the major source of serum and hepatic RANTES/CCL5 in a process mediated by the cellular accumulation of lipids [62]. Moreover, RANTES/CCL5 is also involved in the progression of hepatic fibrosis in mice [63]. Taken together, these results point to the RANTES-CCR5 pathway as a promising therapeutic target in NAFLD and NASH.

Other chemokines, such as CXCL8, CXCL9, and CXCL10, are additional mediators of NAFLD and NASH [64]. CXCL8, which is produced by several cell types, including inflammatory and endothelial cells, induces neutrophil recruitment within inflammatory tissues. Serum CXCL8 levels are significantly higher in patients with NASH than in those with hepatic steatosis or in healthy controls [64]. CXCL9 and CXCL10 share the common receptor CXCR3, which is highly expressed in activated T-cells and natural killer cells [64]. Serum CXCL9 levels are much higher in patients with than without NASH, which suggests CXCL9/CXCR3 signaling as a target for the treatment of liver fibrosis [65,66].

## 3. Antioxidant Carotenoids for the Treatment of NAFLD

Despite the pivotal role of immune cell infiltration in obesity-associated insulin resistance and metabolic diseases such as NAFLD and NASH, there is no consensus on the most effective pharmacological agents for the treatment of either condition, because their pathologies are not fully understood.

Micronutrient antioxidants, such as vitamins and carotenoids, are mainly found in fruits and vegetables and they protect against the formation of reactive oxygen species [67]. Low antioxidant levels are present in the serum and liver tissue of patients with chronic liver diseases [68] and are associated with liver dysfunction, particularly in the case of carotenoids [69]. Thus, micronutrient antioxidant deficiencies may contribute to the development of obesity and comorbidities such as insulin resistance and NASH [70,71]. Although carotenoids are as potent as vitamin E in inhibiting lipid peroxidation [72], carotenoid supplementation (β-cryptoxanthin and astaxanthin) has not been widely used as an antioxidant treatment for patients with NASH. In two studies, we showed that carotenoids, including β-cryptoxanthin and astaxanthin, exhibit antioxidant and anti-inflammatory effects [67,73,74,75], in addition to regulating M1/M2 macrophage polarization in NASH [76,77].

### 3.1. β-Cryptoxanthin

β-Cryptoxanthin is a xanthophyll carotenoid readily absorbed by the body and relatively abundant in human plasma [78,79,80]. Consistent with its antioxidant activity, serum concentrations of β-cryptoxanthin are inversely related to indices of oxidative DNA damage and lipid peroxidation [81]. Recent epidemiological studies have shown that high levels of serum β-cryptoxanthin are associated with improved insulin resistance and alcoholic liver dysfunction in non-diabetic individuals [79,80]. In addition, in both in vivo and in vitro studies, β-cryptoxanthin was shown to have anti-inflammatory effects that operate, primarily, by modulating the innate immune response induced by macrophages [82]. Of note, the expressions of genes encoding chemokines, including MCP-1, CXCL10, and macrophage inflammatory protein-1α, and pro-inflammatory cytokines, such as TNF-α, IL-1β, and IL-6, are significantly decreased in liver and adipose tissue exposed to β-cryptoxanthin [83]. These findings suggest M1-activated macrophages/Kupffer cells or chemokines as a promising therapeutic target in insulin resistance, metabolic syndrome, and NAFLD.

**Figure 3:**
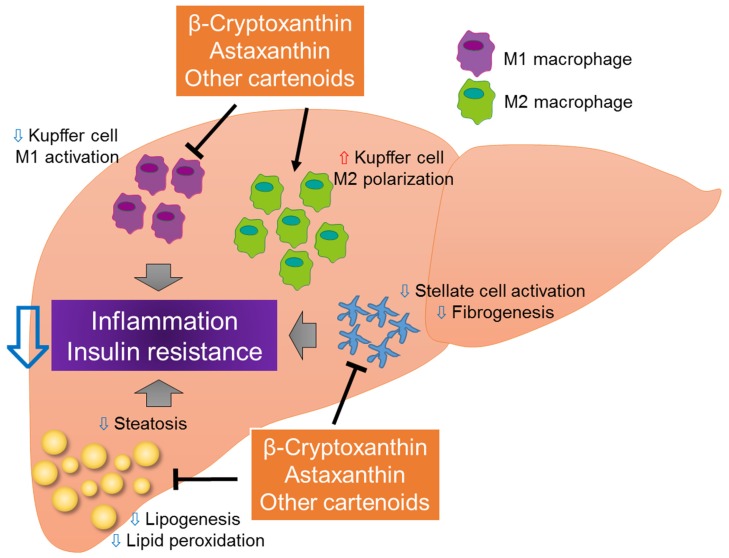
Novel effects of carotenoids on the progression of NASH. Carotenoids, such as β-cryptoxanthin and astaxanthin, may improve NASH by inhibiting lipid accumulation, lipid peroxidation, hepatic inflammation, and hepatic stellate cell activation. By inhibiting M1 macrophage/Kupffer cell activation and inducing a dominant shift of M2 macrophage/Kupffer cell polarization, carotenoids may inhibit hepatic steatosis, inflammation, and insulin resistance.

We previously demonstrated that β-cryptoxanthin prevents the development of NASH by suppressing lipid accumulation, lipid peroxidation, and insulin resistance (Figure 3) [76,84]. Specifically, β-cryptoxanthin prevents the accumulation of T-cells and the activation of hepatic stellate cells, in addition to regulating the M1/M2 status of Kupffer cells in the liver, in part by down-regulating the MCP-1-CCR2 and RANTES-CCR5 pathways [84]. The expression of several genes associated with cell death, inflammation, free radical scavenging, and the recruitment and activation of macrophages/Kupffer cells, leukocytes, and T-cells, is also inhibited by β-cryptoxanthin [84]. Although β-cryptoxanthin does not regulate the expression of genes associated with the metabolism of cholesterol or other lipids, it does reduce hepatic lipid accumulation and peroxidation through its anti-oxidative effects [84]. In addition, β-cryptoxanthin directly decreases LPS-induced M1 macrophage activation and increases IL-4-induced M2 macrophage activation in vitro, suggesting the direct targeting of macrophages by β-cryptoxanthin [76]. Therapeutic strategies that inhibit M1 polarization and/or drive alternative M2 macrophage/Kupffer cell activation may protect against inflammation, thereby halting NASH progression. By reducing the recruitment of pro-inflammatory immune cells and an M2-dominant shift in macrophages/Kupffer cells, β-cryptoxanthin may be a promising therapy for NASH patients.

### 3.2. Astaxanthin

The xanthophyll carotenoid astaxanthin is found in various marine organisms, including salmon, shrimp, and crustaceans, as well as in yeast [85]. The antioxidant effect of astaxanthin is well established; indeed, astaxanthin activity is stronger than that of vitamin E and β-carotene [86]. Furthermore, astaxanthin is 100- to 500-fold more effective than vitamin E in inhibiting lipid peroxidation and it inhibits carbon tetrachloride-induced lipid peroxidation in a rat’s liver. Astaxanthin also prevents the activation of hepatic stellate cells, thereby suppressing the up-regulation of fibrogenic genes by blocking transforming growth factor-β/Smad3 signaling [87,88,89]. In addition, it interferes with diet-induced obesity and hepatic lipid accumulation in mice, and ameliorates oxidative-stress-induced insulin resistance through the enhancement of insulin signaling and the inhibition of pro-inflammatory signaling [90,91,92].

In a previous study, we compared the preventative and therapeutic effects of astaxanthin and vitamin E in a mouse model of NASH [77]. Astaxanthin was more effective than vitamin E in attenuating insulin resistance, hepatic lipid accumulation and peroxidation, liver inflammation, and fibrosis [77]. For instance, astaxanthin decreased the concentrations of triglyceride, total cholesterol, nonesterified fatty acids, ALT, and aspartate aminotransferase. It also prevented the transformation of simple steatosis to NASH in obese mice. Of note, astaxanthin inhibited the activation of the Jun N-terminal kinase/p38 mitogen-activated protein kinases signaling pathway, as well as the nuclear factor-κB pathway, by inhibiting the recruitment and activation of T-cells and macrophages/Kupffer cells. It also induced an M2-dominant shift in macrophages/Kupffer cells to improve inflammation and insulin sensitivity. In an in vitro study, we showed that astaxanthin acts directly on hepatocytes, decreasing lipid accumulation, improving insulin signaling, and inhibiting pro-inflammatory signaling. Astaxanthin administration also decreased M1 macrophage activation and increased M2 macrophage activation in RAW264.7 cells, indicating its direct action on macrophages/Kupffer cells. Given these beneficial effects, astaxanthin should be further evaluated as a novel and promising therapy for NASH (Figure 3).

### 3.3. Other Carotenoids and Other Therapeutic Approach to NASH

Vitamins and carotenoids other than β-cryptoxanthin or astaxanthin also seem to protect against the development of NAFLD. For instance, vitamin B_12_, which is stored in the liver, is associated with the regulation of adipocyte hypertrophy and type 2 diabetes [93]. Lycopene, a non-provitamin carotenoid, is contained in red fruits and vegetables such as tomatoes, red grapefruit, watermelon, and apricots. The administration of lycopene reduces the risk of cancer in many organs and prevents the development of other diseases, including hepatocarcinogenesis linked to nonalcoholic steatohepatitis [94]. Lycopene also inhibits the development of high-fat-diet-induced hepatic steatosis [95]. The significant decrease in the plasma lycopene levels of NASH patients indicates a relationship between a low-lycopene state and the development of liver diseases, including NASH [96].

β-Carotene is the most widely distributed carotenoid. It is mainly found in yellow-orange and dark green fruits and vegetables. Recent studies have demonstrated the potential preventive and protective effects of β-carotene on hepatic steatosis, fibrosis, oxidative stress, inflammation, and apoptosis [67]. Thus, dietary β-carotene supplementation should be considered to prevent the initiation and progression of NASH.

Recent report have shown that Mediterranean diet, which was recognized as a healthy diet, is effective in reducing the risk of cardiovascular disease and cancer [97]. Furthermore, it has been also reported that a Mediterranean diet is an effective non-pharmaceutical option for type 2 diabetes and obesity [98,99]. Moreover, Silymarin, which is the extracts of milk thistle, has been used for the prevention of liver fibrosis by regulating the anti-fibrogenic and anti-inflammatory function [100]. Silymarin treatment is associated with a reduction of insulin resistance and an improvement in liver function [101,102]. Therefore, a dietetic regimen should also be considered to prevent the progression to NASH and its associated metabolic diseases.

## 4. Conclusions and Perspectives

NAFLD has become one of the most common liver diseases worldwide. Its complex pathogenesis is closely associated with disorders such as obesity, type 2 diabetes mellitus, and insulin resistance, and with the progression to hepatic steatosis, fibrosis, and cirrhosis. Several lines of evidence suggest that increased oxidative stress and changes in several molecular factors, including adipokines, chemokines, and pro- or anti-inflammatory cytokines, are mainly involved in the progression of NAFLD to NASH. Macrophages/Kupffer cells play a central role in the pathogenesis of oxidative stress, insulin resistance, and NAFLD. Carotenoids, which are natural compounds with strong antioxidant and anti-inflammatory effects, can inhibit hepatic steatosis, inflammation, and fibrosis. Accordingly, they may be effective in the prevention and treatment of NAFLD. Our recent research has shown that the dietary administration of β-cryptoxanthin or astaxanthin not only prevents but also reverses NASH progression in mice, by regulating M1/M2 macrophage/Kupffer cell polarization. However, there is no evidence that these carotenoids exhibit beneficial effects against the patients with NAFLD. Furthermore, there are no validated non-invasive biomarkers for the therapeutic effects of β-cryptoxanthin or astaxanthin. Currently, biopsy is the only clinical tool available for the diagnosis of pathological alterations in the liver [103]. Future studies are warranted to demonstrate the more detailed mechanisms underlying the pathogenesis of NAFLD and the potential effect of carotenoids in the prevention and treatment of this increasingly widespread disease.
